# Sleep Apnea and Heart Failure—Current State-of-The-Art

**DOI:** 10.3390/ijms25105251

**Published:** 2024-05-11

**Authors:** Tushar Menon, Dinesh K. Kalra

**Affiliations:** 1 Division of Cardiology, University of Louisville Hospital, 201 Abraham Flexner Way, Suite 600, Louisville, KY 40202, USA; 2 Lipid Clinic & Infiltrative Heart Disease Program, Rudd Heart & Lung Center, Division of Cardiovascular Medicine, Department of Medicine, University of Louisville School of Medicine, 201 Abraham Flexner Way, Suite 600, Louisville, KY 40202, USA

**Keywords:** sleep-disordered breathing (SDB), obstructive sleep apnea (OSA), central sleep apnea (CSA), heart failure (HF), cardiac remodeling (CR), cardiovascular outcomes (COs), central positive airway pressure (CPAP), adaptive servo-ventilation (ASV)

## Abstract

Sleep-disordered breathing (SDB), including obstructive and central sleep apnea, significantly exacerbates heart failure (HF) through adverse cardiovascular mechanisms. This review aims to synthesize existing literature to clarify the relationship between SDB and HF, focusing on the pathophysiological mechanisms, diagnostic challenges, and the effectiveness of treatment modalities like continuous positive airway pressure (CPAP) and adaptive servo-ventilation ASV. We analyzed peer-reviewed articles from 2003 to 2024 sourced from PubMed, EMBASE, Scopus, and Web of Science databases. The prevalence of SDB in HF patients is high, often underdiagnosed, and underappreciated. Management strategies, including CPAP and ASV, have been shown to mitigate symptoms and improve cardiac function. However, despite the availability of effective treatments, significant challenges in screening and diagnosis persist, affecting patient management and outcomes. DB significantly impacts HF prognosis. Enhanced screening strategies and broader utilization of therapeutic interventions like CPAP and ASV are essential to improve the management and outcomes of HF patients with concomitant SDB. Future research should focus on refining diagnostic and treatment protocols to optimize care for HF patients with SDB.

## 1. Introduction

Recent studies demonstrate a link between SDB and HF. SDB encompasses obstructive sleep apnea (OSA) and central sleep apnea (CSA) and is common in HF patients, affecting 50–80%, 47–76% in patients who have HF with reduced ejection fraction (HFrEF) and 55% in HF with preserved ejection fraction (HFpEF). [1]. The Sleep-Disordered Breathing in Heart Failure (SchlaHF) registry has revealed that nearly half of patients with chronic stable HFrEF experience moderate to severe SDB. Despite its prevalence, SDB remains underdiagnosed and underestimated, often due to a lack of awareness among both patients and physicians. It is estimated that about one billion individuals worldwide are affected by obstructive sleep apnea-hypopnea syndrome (OSAHS), highlighting the scale of this issue [2].

Patients with HF exhibiting SDB often face a complex symptomatology that includes frequent nighttime apneic episodes and awakenings that disrupt sleep patterns and decrease sleep quality. During the day, these individuals may suffer from excessive daytime sleepiness, fatigue, and cognitive issues like impaired concentration and memory lapses. This fragmented sleep exacerbates the underlying HF condition by causing significant fluctuations in intrathoracic pressure, oxygen desaturation, and sympathetic nervous system activation, worsening cardiac symptoms [3]. Furthermore, sustained sympathetic activation and potential endothelial dysfunction from fragmented sleep increase the risk of hypertension and arrhythmias [3]. SDB significantly diminishes the health-related quality of life in HF; however, effective management can improve the quality of life and HF outcomes. Frequent underdiagnosis of SDB in HF patients indicates a substantial gap in awareness and diagnosis and, therefore, requires the development and implementation of effective screening, diagnostic, and therapeutic strategies. This review aims to comprehensively analyze the pathophysiological links and risk factors between sleep-disordered breathing and heart failure, critically assess the efficacy and limitations of current treatment strategies, and suggest directions for future research to optimize therapeutic outcomes.

## 2. Methods

We conducted a comprehensive narrative review to investigate the relationship between SDB and HF, sourcing literature from 2003 to 2024. Our search encompassed several databases, including PubMed, EMBASE, Scopus, and Web of Science. Keywords related to SDB, such as “obstructive sleep apnea” (OSA) and “central sleep apnea” (CSA), were paired with terms pertinent to HF to ensure a targeted retrieval of relevant studies. We restricted our review to peer-reviewed articles written in English, focusing on those that explored pathophysiological mechanisms, diagnostic approaches, and treatment outcomes related to the interplay between SDB and HF. Exclusion criteria included letters to the editor, unpublished manuscripts, and abstracts from conferences, ensuring the credibility and reliability of our sources. The narrative review also carefully selected seminal works and authoritative reports to provide a historical context and foundational understanding of the complex relationship between these conditions. Priority was given to studies published in the past decade to capture the most recent scientific advancements. However, pivotal earlier works were also considered to trace the evolution of knowledge in this field.

## 3. Introduction to Obstructive and Central Sleep Apnea

### 3.1. Obstructive Sleep Apnea

OSA is characterized by a recurrent collapse of the pharyngeal airway during sleep due to an anatomical narrowing and increased compliance of the pharynx, which is often exacerbated by reduced pharyngeal dilator muscle tone accompanying sleep onset [4]. The efforts to breathe against an occluded airway lead to substantial fluctuations in intrathoracic pressure, which imposes increased afterload on the left ventricle and disrupts venous return to the heart [4]. This disruption leads to a cascade of cardiovascular responses, including heightened sympathetic nervous system activity due to the cessation of the inhibitory influence of pulmonary stretch receptors and the stimulatory effects of hypoxia and hypercapnia on peripheral and central chemoreceptors [4].

### 3.2. Central Sleep Apnea

CSA associated with Cheyne–Stokes respiration represents a form of periodic breathing marked by a characteristic waxing–waning pattern in tidal volume alternating between hypopneas and periods of hyperventilation [5]. The pathophysiology of CSA involves several interconnected mechanisms. Most heart failure patients with CSA experience chronic hyperventilation due to stimulation of pulmonary vagal irritant receptors through pulmonary congestion and enhanced chemosensitivity [5]. This hyperventilation, exacerbated when patients lie flat due to increased venous return and subsequent pulmonary congestion, reduces PaCO_2_ below the threshold required to stimulate breathing, thus initiating apnea [5]. The apnea persists until PaCO_2_ rises above the threshold to resume ventilation.

While the association between CSA and congestive heart failure (CHF) is well recognized, the precise nature of their connection remains ambiguous. CHF may predispose individuals to CSA, and CSA, in turn, may exacerbate CHF progression. One aspect of this interaction is elevated sympathetic nerve activity, marked by increased urinary and plasma norepinephrine levels and potentially enhanced endothelin levels, associated with poorer prognoses in CHF [6]. Moreover, the presence of CSA in patients with asymptomatic left ventricular dysfunction signifies an early disruption in cardiac autonomic regulation, increasing the risk of cardiac arrhythmias before overt HF symptoms manifest [6].

**Figure 1:**
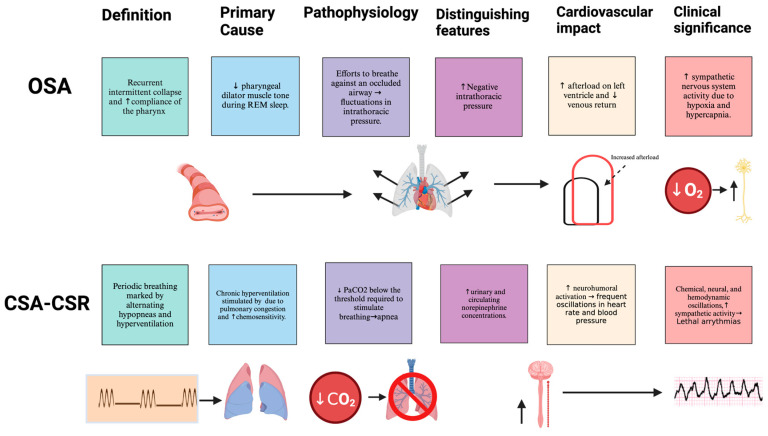
OSA is characterized by recurrent intermittent pharyngeal airway collapse during sleep, primarily due to anatomical narrowing and decreased muscle tone, leading to mechanical and hemodynamic changes. The condition emphasizes the negative intrathoracic pressure and systemic cardiovascular responses triggered by hypoxia and hypercapnia. Conversely, CSA-CSR involves periodic breathing patterns and is often associated with HF, driven by chronic hyperventilation, pulmonary congestion, and enhanced chemosensitivity. It lacks the negative intrathoracic pressure seen in OSA but shows increased norepinephrine levels, contributing to neurohumoral activation, fluctuations in blood pressure and heart rate, and a higher risk of lethal arrhythmias.

In clinical practice, CSA in CHF often coincides with reduced exercise capacity, lower ejection fractions, larger left ventricular volumes, and increased pulmonary capillary wedge pressure, collectively suggesting a more advanced form of CHF [4]. This implies that CSA may serve as both an early indicator and a potential contributor to the progression of advanced CHF (Figure 1). 

## 4. Risk Factors and Demographics of Sleep Apnea and Heart Failure

**Figure 2:**
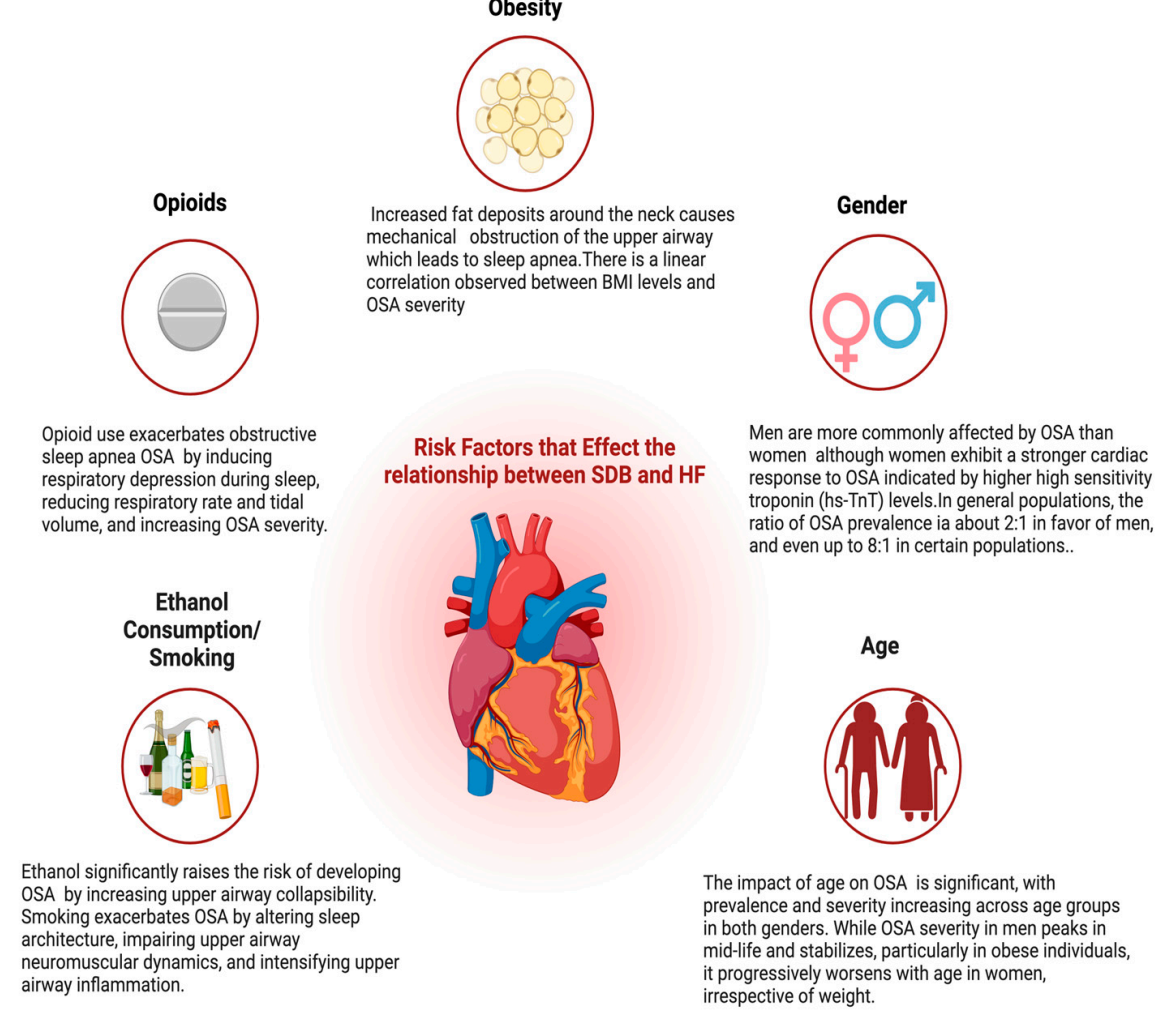
Risk factors such as obesity, gender differences, smoking, alcohol consumption, and opioid use are crucial in linking OSA and HF. Obesity, marked by increased fat deposits around the neck, leads to airway obstruction and sleep apnea, escalating cardiovascular strain. Opioid use exacerbates these conditions by reducing respiratory rate and tidal volume. Lifestyle choices like smoking and heavy alcohol consumption further worsen both OSA and HF by promoting inflammation and airway obstruction. The prevalence of OSA increases with age in both genders. Although men are more commonly affected by OSA, women experience more severe cardiac responses.

Recent research has explored relationships between cardiovascular risk factors and OSA, emphasizing their coexistence and the implications for disease severity and management. One such study, “Sleep Disorders Assessed by Polysomnography and Non-invasively Estimated Risk of Significant Coronary Artery Disease in the Population of Patients with Arterial Hypertension,” involved 88 individuals aged 53.76 ± 12.59 years and utilized comprehensive diagnostic tools including questionnaires, anthropometric measurements, blood tests, and polysomnography to assess the interplay between various cardiovascular risk factors (CVRFs) and the incidence and severity of OSA. Notably, the research revealed a directly proportional relationship between the number of CVRFs present and the severity of OSA [7]. This correlation is critical as it underscores the synergistic effect of each additional risk factor on exacerbating sleep apnea severity. Additionally, obesity, arterial hypertension, and smoking were found to be independent predictors of higher AHI values [7]. These insights are pivotal for understanding the risk factors linking OSA with HF and guiding effective clinical interventions (Figure 2).

### 4.1. Obesity

Obesity is a crucial risk factor for OSA, particularly in men. An elevated body mass index (BMI) leads to the narrowing of the airway lumen, significantly heightening the likelihood of OSA. This results in repeated episodes of partial or complete airway closure during sleep [8]. These recurring episodes in moderate-to-severe OSA cases trigger acute physiological stresses, including arterial oxygen desaturation, surges in sympathetic activity, and acute hypertension, leading to further worsening of HF [8].

### 4.2. Ethanol Consumption/Smoking

The relationship between OSA and HF is linked to lifestyle factors such as smoking and alcohol consumption. Notably, alcohol significantly influences the risk of developing OSA, which, in turn, can exacerbate heart failure. A systematic review and meta-analysis that pooled data from 21 studies showed a 25% increase in the risk of sleep apnea associated with higher alcohol consumption (RR 1.25, 95% CI (1.13–1.38, *p* < 0.0001), with alcohol likely to increase upper airway collapsibility and thereby aggravating sleep apnea symptoms [5,9]. This increased risk persisted across different definitions of alcohol consumption and despite adjustments for various confounders such as body mass index and smoking [9]. Smoking exerts multifactorial influences on the pathophysiology of OSA, thereby augmenting the risk and severity of HF. The pathogenetic mechanisms include alterations in sleep architecture, impairment of upper airway neuromuscular dynamics, modulation of arousal thresholds, and exacerbation of upper airway inflammation [10]. These smoking-related alterations in OSA can lead to significant cardiovascular complications, further escalating the risk of heart failure.

### 4.3. Opioid Use

Opioid use has been increasingly identified as a significant risk factor for both the development and exacerbation of OSA. These conditions critically influence the management and outcomes of heart failure. Opioids induce dose-dependent respiratory depression, more pronounced during sleep, which leads to decreased respiratory rates and tidal volumes. As a result, this respiratory depression can aggravate existing sleep-disordered breathing and potentially heighten the severity of OSA. Detailed insights from a systematic review and meta-analysis shed light on the quantifiable impacts of opioid use on OSA. The review included 15 studies, comprising six clinical trials and nine observational studies, which were scrutinized to determine the effects of opioids on OSA severity [11]. The meta-analysis, which included ten of these studies, found a pooled mean change AHI of 1.47 per hour associated with opioid use, with a confidence interval ranging from −2.63 to 5.57 (*p* = 0.002) [11]. This finding suggests a modest but variable impact of opioids on increasing AHI, indicative of worsened OSA symptoms. Furthermore, the review highlighted that interventions such as continuous positive airway pressure (CPAP) were generally less effective in opioid users, with higher failure rates and reduced efficacy in reducing AHI compared with non-opioid users [11].

### 4.4. Age

The influence of age on OSA is profound and varies significantly across different age groups. Data from a large cohort of 23,806 patients who underwent whole-night polysomnography showed that the prevalence and severity of OSA increased with age in both men and women. However, the patterns and implications of these increases differed between genders. For women, the severity of OSA, as measured by AHI, increased linearly with age [12]. This trend was maintained regardless of whether they were average weight or obese, highlighting age as a critical factor in the progression of OSA among women [12]. In men, however, it was reported that the severity of OSA increased significantly in the early adult years up to mid-life, particularly among obese individuals, after which it tended to stabilize [12]. This suggests that while age is a significant factor in the escalation of OSA severity for both genders, the impact is more pronounced and occurs earlier in life for men, particularly those who are obese.

Moreover, the age-related increase in AHI also corresponds with a shift in the clinical presentation and the predictive value of various symptoms and comorbid conditions. For instance, the cutoff points for AHI that best predict OSA shift higher as age increases [12]. This shift in diagnostic thresholds with age underscores the need for clinicians to consider age when evaluating symptoms and diagnostic test results.

### 4.5. Gender

Obstructive sleep apnea (OSA) disproportionately affects men in comparison to women, with a prevalence ratio of 2:1 in population-based studies, which can escalate to 8:1 in specific demographics [13]. Notably, men generally have higher incidences of both OSA and hypertension. However, the implications of OSA severity appear more pronounced in women, as it is strongly correlated with elevated levels of high-sensitivity troponin T (hs-TnT), suggesting a heightened risk of heart failure (HF) [13]. This gender-specific variation implies that women may experience a more severe cardiac response to the intermittent hypoxemic episodes characteristic of OSA. Research further substantiates that OSA’s association with hs-TnT is exclusive to women, showing no independent correlation in men [13]. Likewise, OSA is connected to an increased risk of heart failure or death in women, a link that diminishes upon adjusting for hs-TnT levels, suggesting that hs-TnT may mediate this relationship [13]. Additionally, in women, OSA severity measured in midlife independently predicts a higher left ventricular mass index later in life, a relationship not observed in men [13]. These findings underscore significant sex-specific differences in the cardiovascular impact of OSA, highlighting the critical need to incorporate a gender-focused approach in both the clinical management of OSA-related health risks and associated research.

## 5. Cardiac Implications of Sleep Apnea

### 5.1. Fluid Balance

In patients with OSA, dysregulated fluid homeostasis significantly impacts upper airway patency through mechanisms mediated by nocturnal fluid redistribution. Particularly in fluid-retaining states such as heart failure, elevated venous pressure during periods of recumbency facilitates a rostral fluid shift from the lower extremities toward the neck [14]. This redistribution is exacerbated by the horizontal posture during sleep, leading to an increased volume and pressure in the cervical soft tissues. The fluid accumulation in the peri pharyngeal tissues mechanically increases external pressure exerted on the pharyngeal walls, reducing the upper airway’s cross-sectional area [14]. This mechanical load on the airway increases its collapsibility. It heightens the likelihood of complete obstruction during sleep, particularly during the inspiratory phase when negative intrathoracic pressure peaks [14]. Moreover, this fluid shift is not uniformly distributed. It may disproportionately affect individuals with higher degrees of cardiovascular compromise or those with compromised lymphatic drainage systems, who are often unable to counteract the effects of increased hydrostatic pressure [14].

### 5.2. Loop Gain

Loop gain, an engineering concept, is crucial in understanding the pathophysiology of SDB, especially CSA in heart failure. It measures the respiratory control system’s sensitivity to ventilation disturbances, indicating how small changes in blood gases can destabilize breathing patterns and lead to cycles of apnea and hyperventilation [15]. Comprising controller gain, plant gain, and mixing gain, it influences the stability of the respiratory system [15]. Controller gain is linked to chemoreceptor sensitivity to blood gas changes, potentially causing exaggerated ventilatory responses [15]. Plant gain evaluates how efficiently the respiratory system clears CO_2_; higher efficiency can paradoxically destabilize respiratory patterns by too rapidly adjusting CO_2_ levels [15]. Mixing gain, influenced by the circulation time, is often prolonged in heart failure, further destabilizing breathing [15]. High loop gain in heart failure patients usually results in Cheyne––Stokes respiration, leading to severe nocturnal oxygen drops, intrathoracic pressure fluctuations, and increased sympathetic activity, which can worsen heart failure and increase the risk of myocardial ischemia and arrhythmias [15].

### 5.3. Intrathoracic Pressure Dynamics

**Figure 3:**
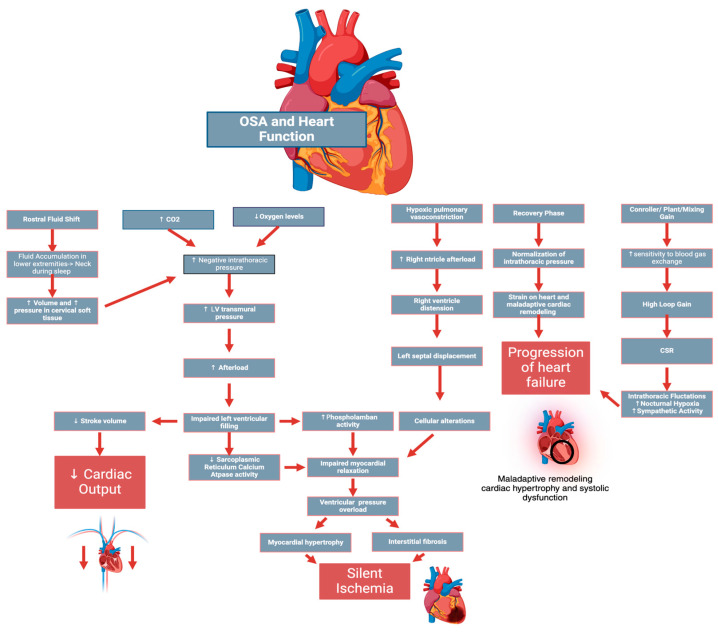
Pathophysiological mechanisms by which obstructive sleep apnea (OSA) affects cardiac function include increased CO_2_ levels, drop in oxygen levels, and resulting in hypoxic pulmonary vasoconstriction. The figure shows how increased negative intrathoracic pressure and its effects on left ventricular transmural pressure lead to increased afterload and reduced cardiac input. The normalization of intrathoracic pressure and subsequent right ventricle distension are highlighted, illustrating the strain on the heart and the potential for maladaptive cardiac remodeling resulting in the progression of heart failure. It also covers left septal displacement, resulting in alterations at a cellular level that contribute to impaired myocardial relaxation and silent ischemia. It touches on how increased phospholamban activity and decreased sarcoplasmic reticulum calcium ATPase activity contribute to silent ischemia. It connects increased pressure in the ventricles to myocardial hypertrophy and interstitial fibrosis, the two major driving forces behind silent ischemia. It also indicates how fluid balance is profoundly impacted by nocturnal rostral fluid shifts toward the neck, increasing airway compressibility, negative intrathoracic pressure, and cardiac workload. It further explores how high loop gain, related to sensitivity to blood gas changes, can exacerbate the cyclical breathing pattern in OSA, compounding cardiovascular stress and facilitating the progression of heart failure.

During apneic episodes, the cessation of breathing leads to a build-up of carbon dioxide and a subsequent drop in oxygen levels, triggering a series of cardiovascular responses. The negative intrathoracic pressure generated against the occluded airway during attempts to breathe increases left ventricular transmural pressure, significantly elevating afterload [16]. Moreover, the negative intrathoracic pressure impacts right ventricular function by enhancing venous return during apnea, leading to right ventricular overload and distension that adversely affect left ventricular filling due to ventricular interdependence. This combination impairs LV filling, reducing stroke volume and cardiac output [16]. Concurrently, OSA-induced hypoxic pulmonary vasoconstriction increases right ventricular afterload, leading to RV distension and leftward septal displacement during diastole, thereby exacerbating the impact on cardiac function [16]. This overload induces cellular alterations, such as decreased sarcoplasmic reticulum calcium ATPase (SERCA) pump activity, resulting in inadequate calcium sequestration in the sarcoplasmic reticulum of cardiac myocytes, disrupting the myocardial relaxation phase post-contraction [17]. Concurrently, heightened phospholamban activity synergistically impedes SERCA function due to reduced phosphorylation, exacerbating intracellular calcium imbalance and precipitating diastolic dysfunction [17]. Ventricular pressure overload also stimulates pathways leading to myocardial tissue hypertrophy and interstitial fibrosis, increasing passive stiffness and causing silent ischemia, further impairing ventricular relaxation [17]. The recovery phase following apneic episodes presents its own challenges. The resumption of normal breathing leads to a rapid normalization of intrathoracic pressure, and this sudden shift can strain the left ventricle as it adapts to rapidly changing hemodynamic conditions [16]. Over time, these recurrent cycles of exaggerated negative intrathoracic pressure and subsequent recovery can lead to maladaptive cardiac remodeling (Figure 3) [16]. Notably, even though the elimination of apneic events through CPAP therapy can partially reverse these alterations, the structural changes in the heart may not be fully reversible, highlighting the impact of untreated OSA [18].

### 5.4. Intermittent Hypoxia

Intermittent hypoxia, characterized by short, repetitive cycles of hypoxia and reoxygenation, plays a critical role in the pathogenesis of systemic inflammation and subsequent cardiac stress. This relationship is mediated through the activation of key transcription factors, notably hypoxia-inducible factor-1 (HIF-1) and nuclear factor-kB (NF-kB), which are integral to the inflammatory repercussions of OSA [19]. The process begins with intermittent hypoxia-stimulating cytokine production and activating inflammatory cells, leading to vascular changes. The influence of intermittent hypoxia on NF-kB activity and its downstream products, like TNF-α, directly relates to the extent of cardiovascular damage through the expression of various inflammatory agents, including cytokines, chemokines, and adhesion molecules [19]. 

Simultaneously, HIF-1, the primary regulator of cellular oxygen homeostasis, balances responses to hypoxia by increasing tissue perfusion and oxygenation [19]. However, this mechanism has a paradoxical effect; HIF-1 also prolongs the survival of myeloid inflammatory cells, thereby prolonging inflammation and exacerbating cardiac stress [19]. Patients with OSA have also been shown to have increased levels of other inflammatory markers, such as interleukin 6 (IL-6) and C-reactive protein (CRP), which play significant roles in the inflammatory pathways activated by intermittent hypoxia [20]. CRP is believed to play a direct role in the development of vascular disease via contributing to atherosclerosis through inhibiting the activity of nitric oxide synthase and promoting increased expression of cell adhesion molecules [20]. The interaction between these pathways demonstrates a complex regulatory mechanism in response to intermittent hypoxia. 

Intermittent hypoxia enhances the sensitivity of carotid chemoreceptors through several mechanisms. Firstly, it induces oxidative stress, leading to the production of angiotensin-II and endothelin-1 [21]. Angiotensin-II inhibits nitric oxide (NO), a known inhibitor of carotid body activity [21]. Additionally, endothelin-1 stimulates carotid chemoreceptors and acts as a vasoconstrictor peptide, contributing to vascular restructuring and elevating blood pressure [21]. Over time, these responses may contribute to the progression of cardiovascular diseases, including heart failure. In individuals with heart failure, the heightened sensitivity of carotid chemoreceptors can exacerbate the already compromised cardiovascular function, potentially worsening symptoms and outcomes.

### 5.5. Adrenergic Response

In contrast to OSA, CSA does not generate negative intrathoracic pressure during apnea [5]. The focus in CSA has been on its adrenergic effects as the primary mechanism for disease progression. Patients with HF and CSA exhibit higher urinary and circulating norepinephrine concentrations, both during sleep and wakefulness, compared with HF patients without sleep-related breathing disorders [5]. The magnitude of these increases correlates with the frequency of sleep arousals and the degree of apnea-related hypoxia. CSA also induces very low-frequency oscillations in ventilation, causing the heart rate to oscillate with breathing. This effect on heart rate due to periodic breathing leads to a shift in heart rate from predominantly high frequency during regular breathing to shallow frequency [5]. Although these synchronized oscillations minimize ventilation/perfusion mismatch, CSA is associated with a higher mortality rate in patients with HF [5].

The activation of the sympathetic nervous system in patients with SDB and heart failure contributes to cardiovascular stress. SDB stimulates autonomic reflex responses via activating peripheral and central chemoreceptors. This stimulation increases muscle sympathetic nerve activity (MSNA) and vasoconstriction, particularly in patients with heart failure and systolic dysfunction [22]. Despite the increased sympathetic activity, the anticipated decrease in muscle blood flow is not observed, suggesting a complex interplay between sympathetic outflow and vascular response mechanisms [22]. This response results in altered hemodynamics, leading to an increased risk of cardiovascular morbidity and mortality in this patient population.

## 6. OSA and Cardiac Arrhythmogenesis

### 6.1. Atrial Fibrillation

**Figure 4:**
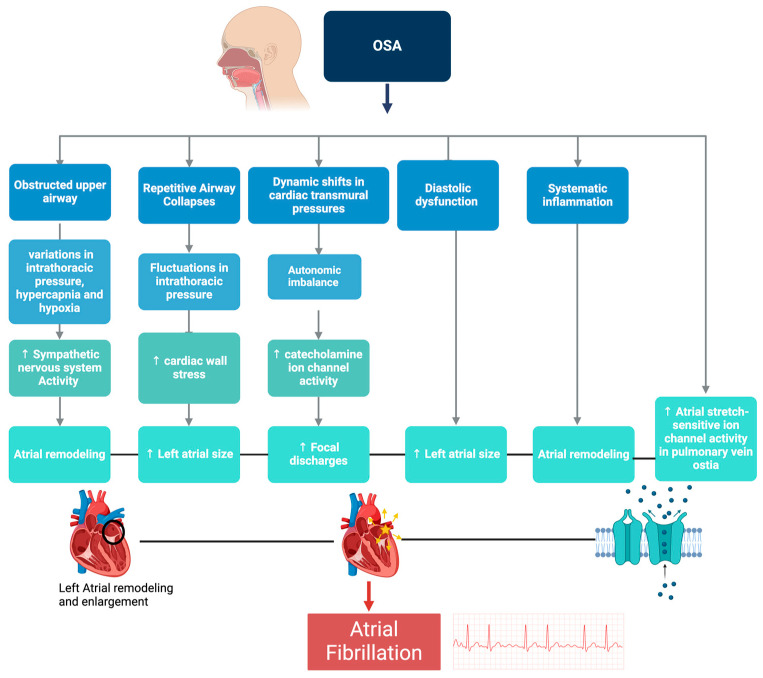
Relationship between obstructive sleep apnea (OSA) and atrial fibrillation (AF). Hypoxia-induced sympathetic activation, fluctuations in intrathoracic pressure, and the subsequent autonomic imbalance coupled with diastolic dysfunction and atrial remodeling set the stage for AF. The figure also depicts the mechanisms by which systemic inflammation and activation of stretch-sensitive ion channels further predispose patients with OSA to AF.

The enhanced sympathetic nervous system activity observed in SDB and HF drives the pathophysiological connection between OSA and atrial fibrillation (AF). Repetitive episodes of upper airway obstruction during sleep lead to cyclic variations in intrathoracic pressure, intermittent hypoxemia, and hypercapnia. These physiological disturbances trigger sympathetic nervous system activation and oxidative stress, contributing to atrial remodeling [22]. Diastolic dysfunction is also independently associated with OSA and AF. The alteration in mitral inflow patterns, reflected in the E-A ratio and deceleration time, correlates with the severity of nocturnal oxygen desaturation [23]. This can lead to increased left atrial size, a critical factor in the development of AF [23]. Concurrently, repetitive Mueller maneuvers, characteristic of OSA, induce wide fluctuations in negative intrathoracic pressure [23]. These changes in cardiac transmural pressure and wall stress further exacerbate left atrial enlargement, activating atrial stretch-sensitive ion channels, particularly in anatomically susceptible areas such as the pulmonary vein ostia, which can initiate AF [23]. The dynamic shifts in cardiac transmural pressures during OSA episodes, severe sympathetic activity surges, and subsequent vagal predominance contribute to a marked autonomic imbalance. This imbalance activates atrial catecholamine-sensitive ion channels, leading to focal discharges, and alters atrial conduction properties, facilitating AF [23]. Additionally, systemic inflammation associated with OSA may further exacerbate the risk of AF through its effects on atrial myocardial remodeling [23]. This complex interplay between OSA-induced systemic and atrial-specific pathophysiological changes (Figure 4) underscores recognizing and managing OSA as a critical modifiable risk factor in AF.

### 6.2. Ventricular Arrhythmias

**Figure 5:**
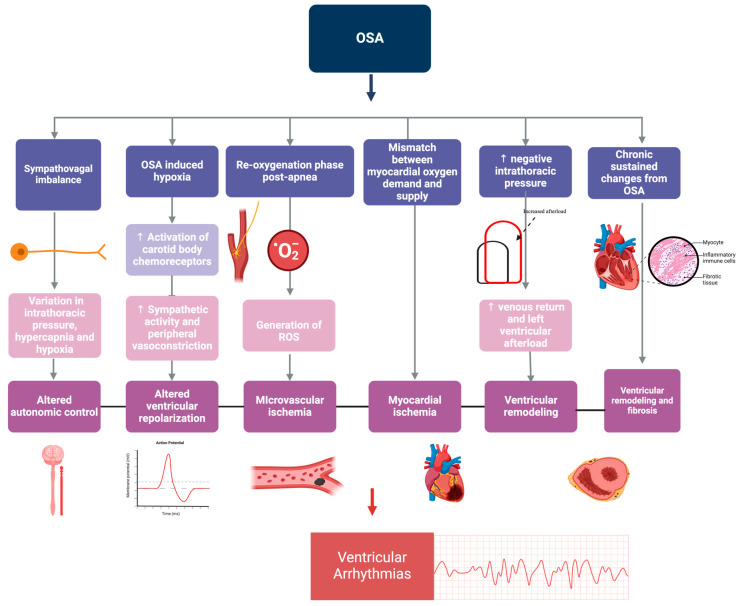
Pathophysiological mechanisms linking obstructive sleep apnea (OSA) to ventricular arrhythmias highlighting sympathovagal imbalance, cyclical variations in intrathoracic pressure, and the impact of hypoxia and re-oxygenation phases. The figure details the mismatch between myocardial oxygen demand and supply, the role of increased negative intrathoracic pressure, and the activation of carotid body chemoreceptors leading to sympathetic activity. It also shows how generating reactive oxygen species, altered ventricular repolarization, microvascular ischemia, ventricular remodeling, and fibrosis all culminate in increased risks of ventricular arrhythmias in patients with OSA.

Sleep apnea is caused by a physiological imbalance, where vagal output increases during apneic episodes, counteracted by a surge in sympathetic activity post-apnea due to hypoxia and hypercapnia. This affects autonomic control and can lead to ventricular arrhythmias [24]. Concurrently, OSA-induced hypoxia activates carotid body chemoreceptors, which enhance sympathetic nerve activity and peripheral vasoconstriction, resulting in increased dispersion of ventricular repolarization and increased expression of left ventricle endocardial calcium channels [24]. The re-oxygenation phase after apnea is characterized by the generation of reactive oxygen species (ROS), further impacting arrhythmogenesis via promoting microvascular ischemia [24]. OSA also results in a pronounced increase in negative intrathoracic pressures during apneic phases, which results in increased venous return and left ventricle afterload, which results in ventricular remodeling [24]. This is exacerbated by myocardial ischemia, which results from the mismatch between increased myocardial oxygen consumption from the mechanisms described and decreased oxygen supply. This is further complicated by the chronic manifestations of OSA, such as ventricular remodeling and fibrosis from sustained alterations in heart rate, blood pressure, increased negative intrathoracic pressure, and sympathetic nerve activity [24]. This constellation of autonomic, hemodynamic, and structural alterations emphasizes the multifaceted influence of OSA on ventricular arrhythmias (Figure 5).

## 7. The Role of CPAP and Adaptive Servo-Ventilation in Sleep Apnea Management

### 7.1. Efficacy of CPAP in Cardiac Function and Symptom Improvement

Continuous positive airway pressure (CPAP) therapy plays a pivotal role in alleviating symptoms of OSA and improving cardiac function in HF patients, particularly those with reduced ejection fraction. Patients with OSA and HF often experience increased metabolic demand and sympathetic nervous system overactivation secondary to norepinephrine release in response to increased stress. In a randomized study involving 45 patients with heart failure and OSA, the effects of short-term CPAP treatment on myocardial sympathetic nerve function were examined [25]. In patients who were assessed via echocardiography and positron emission tomography using 11C-acetate and 11C-hydroxyephedrine before and around 6 to 8 weeks after randomization to either short-term CPAP or no CPAP, it was shown that CPAP significantly improved myocardial sympathetic nerve function, as evidenced by increased retention of hydroxyephedrine, a presynaptic sympathetic nerve activity marker, suggesting a restoration of the heart’s ability to manage stress responses more efficiently, leading to improved cardiac outcomes [25]. In the study, patients with more severe OSA (AHI > 20 events per hour) also experienced significant improvements in work metabolic index and systolic blood pressure, suggesting a potential improvement in cardiac efficiency in this subgroup [25]. In a 2006 study including 43 obese patients with severe OSA and no known cardiac or obstructive pulmonary disease, CPAP treatment for six months led to significant improvements in symptoms, hemodynamics, left and right ventricular morphology, and cardiac function, emphasizing the reversible nature of OSA’s structural and functional consequences for the heart when apneic episodes are effectively managed [26]. 

A comprehensive study by Xu et al. (2021) evaluated the cardiac effects of CPAP therapy in 265 patients with OSA, stratified by obesity status [27]. A significant difference was observed in left ventricular mass and end-diastolic volume ratio across non-obese and obese OSA patients compared with non-OSA individuals [27]. The baseline NT pro-BNP showed no significant differences across groups. However, CPAP therapy reduced NT-proBNP levels in non-obese OSA patients, indicating improved cardiac function [27]. Conversely, in obese OSA patients, CPAP therapy was associated with increased left atrial volume index, with no significant change in NT-proBNP levels [27]. Another study, the Sleep Apnea Cardiovascular Endpoints (SAVE), one of the most extensive multicenter randomized clinical trials assessing the impact of CPAP on cardiovascular outcomes, looked at 2717 patients with moderate to severe OSA and established cardiovascular disease. Patients were randomized to receive CPAP therapy plus usual care or usual care alone. They were followed for 3.7 years for secondary prevention [28]. Despite significantly reducing AHI, CPAP therapy was not associated with a statistically significant reduction in cardiovascular events [28]. However, several factors, such as the exclusion of patients with “sleepy” OSA and severe hypoxemia, the use of automated oximetry data instead of polysomnography for diagnosis, and an overall low adherence rate to CPAP therapy (mean of 3.3 h per night) may have limited the interpretability and generalizability of the findings [28]. These studies highlight the complex interactions between OSA, obesity, and cardiac remodeling and the variable impact of CPAP therapy based on patient-specific factors. Despite the lack of a statistically significant reduction in cardiovascular events in some studies such as SAVE, the evidence supports the conclusion that CPAP therapy, when tailored to patient-specific conditions, especially in those without severe comorbidities such as obesity, can benefit cardiac function. 

### 7.2. Challenges in CPAP Adherence and Its Clinical Implications

Despite its efficacy, many patients find CPAP uncomfortable, leading to poor compliance. A cohort study of 40,485 patients in Denmark diagnosed with sleep apnea from 2000 to 2012 found that only 45.2% adhered to CPAP therapy [29]. Furthermore, within the subset of patients experiencing both sleep apnea and concurrent heart failure, there was no notable variance in adherence to heart failure medication between those who adhered to CPAP therapy and those who did not [29]. This indicates that the challenges in adhering to CPAP do not necessarily translate to adherence to drug therapy [13]. In a 2016 study published in the *New England Journal of Medicine*, researchers explored the impact of CPAP therapy on preventing cardiovascular events in patients with moderate-to-severe OSA and existing cardiovascular disease. This multicenter randomized trial enrolled 2717 adults aged 45 to 75 with moderate-to-severe OSA and existing cardiovascular disease [30]. Participants were randomized to receive either CPAP therapy, usual care, or usual care alone [30]. The primary endpoint was a composite of death from cardiovascular causes, myocardial infarction, stroke, or hospitalization for unstable angina, heart failure, or transient ischemic attack [30]. Secondary endpoints included other cardiovascular outcomes, health-related quality of life, snoring symptoms, daytime sleepiness, and mood [30]. In the CPAP group, mean adherence to CPAP therapy was only 3.3 h per night [30]. This highlights the difficulties in adhering to CPAP therapy faced by patients with moderate-to-severe OSA and established cardiovascular disease. Despite the adherence challenges, CPAP therapy showed notable benefits in reducing symptoms of OSA, such as snoring, daytime sleepiness, and enhancing participants’ quality of life and mood [30]. However, there was no significant difference in primary endpoint events between the CPAP group (17.0%) and the usual-care group (15.4%), indicating that CPAP therapy did not prevent cardiovascular events in that patient population [30]. Further research is needed to explore additional therapeutic strategies to improve cardiovascular outcomes in this high-risk population.

### 7.3. OSA and Heart Failure Readmissions: Assessing CPAP’s Role in Reducing Hospitalizations

Emerging research underscores the role of OSA in predicting recurrent HF-related hospital admissions. A comprehensive study by Sommerfeld et al. (2017) demonstrated that patients with concomitant heart failure and OSA had significantly elevated risk of hospital readmission within 30 and 90 days following discharge compared with their counterparts without OSA [31]. This association persisted even after adjustments for various potential confounders, including age, ethnicity, obesity, diabetes, and chronic obstructive pulmonary disease [31]. The study revealed a 30.3% and 57.6% readmission rate within 30 and 90 days, respectively, for patients afflicted with HF and OSA, compared with 19.6% and 36.3% observed in HF patients without OSA [31]. Healthcare workers need to integrate the management of OSA into the overall therapeutic approach for HF patients, particularly in strategizing post-discharge care to reduce the risk of 30- and 90-day readmissions. Effective adherence to CPAP treatment can reduce healthcare utilization and improve patient outcomes. A study encompassing a cohort of 3182 patients diagnosed with OSA and HFrEF, looking at adherence to PAP therapy, was tracked over the first year and subjects were classified based on an adapted US Medicare definition into three distinct groups: 39% (1252 patients) were deemed adherent, having used the PAP device for at least 4 h per night on 70% of nights across all four consecutive 90-day periods within the year; 29% (935 patients) were classified as intermediately adherent, meeting the adherence criteria in at least one but no more than three of the four quarters; and 31% (995 patients) were identified as nonadherent, failing to meet the criteria in any of the quarters [32]. The study outcomes showed that adherent patients encountered significantly fewer composite visits comprising hospitalizations and emergency room visits than their matched nonadherent counterparts, highlighted by a 24% reduction in emergency room visits among the adherent group [32]. Also, composite visit costs were substantially lower for adherent patients at USD 4526, compared with USD 6258 for intermediately adherent patients and USD 6473 for nonadherent patients, showing a cost gradient reflective of the level of adherence [32].

### 7.4. The Effectiveness and Limitations of CPAP in Managing CSA in HF Patients

It becomes evident that while adherence to PAP therapy significantly influences healthcare utilization and expenditure among patients with OSA and HFrEF, the complexity of sleep apnea treatment extends beyond these parameters. The Canadian Continuous Positive Airway Pressure for Central Sleep Apnea and Heart Failure (CANPAP) trial evaluated the impact of CPAP therapy on 258 patients with CSA and HFrEF who were tracked over an average 2-year follow-up period [33]. That trial highlighted the benefits and limitations of sleep apnea management in heart failure patients. The study found that treating CSA with CPAP attenuated CSA and improved nocturnal oxygenation, LVEF, and exercise capacity [33]. However, CPAP failed to suppress AHI values below the trial entry threshold in 43% of patients and did not affect heart transplant-free survival [33]. These studies suggest that while effective sleep apnea management can reduce healthcare resource utilization and improve patient outcomes, challenges remain in achieving optimal adherence and translating these into long-term survival benefits.

### 7.5. Evaluation of Clinical and Behavioral Treatment Strategies for CPAP Adherence

Strategies such as patient education, regular follow-ups, and fixed PAP machines with digital tracking are recommended to improve CPAP adherence. Addressing these barriers and enhancing patient adherence to CPAP therapy is crucial for optimizing its benefits in improving cardiac function and reducing OSA symptoms.

Significant strides have been made in enhancing patient compliance and therapeutic efficacy for CPAP use in patients with OSA and HF. The development of bi-level PAP (BiPAP) and expiratory pressure relief (EPR) devices reflects a concerted effort to reduce the overall airway pressure delivered [34]. BiPAP offers differing pressures for inhalation and exhalation, thereby reducing mean airway pressure and allowing reduced expiratory pressure without compromising airway patency, benefiting patients intolerant of constant pressure and those with severe OSA. EPR technology aimed at reducing airway pressure during early exhalation initially suggested a notable improvement in CPAP adherence in early non-randomized trials, showing an impressive increase of approximately 1.5 h per night in CPAP use [34]. However, subsequent randomized trials did not confirm a statistically significant improvement in adherence, indicating variability in the effectiveness of EPR among individuals. These innovations in PAP technology mark a substantial advancement in personalized treatment for OSA and HF patients. However, they also highlight the ongoing need for rigorous evaluation to understand their long-term health impacts fully.

Incorporation of heated humidification and a wide array of mask designs are now standard CPAP features. Heated humidification optimizes the airflow’s humidity level, effectively reducing mucosal dryness and irritation in the nasal passages, factors known to compromise CPAP compliance [34]. Introducing a wide array of mask options allows a personalized approach to treatment, accommodating anatomical variability among patients. This customization potential is critical for ensuring an optimal seal and minimizing air leaks, which are essential parameters for the efficacy of CPAP therapy [34]. These technological enhancements in CPAP systems are necessary to mitigate compliance barriers and improve therapeutic outcomes and patient satisfaction.

Psychosocial interventions, including motivational interviewing and cognitive behavioral therapy, have shown promise in improving CPAP adherence [34]. However, their long-term efficacy and practicality in routine care remain subjects of ongoing investigation [34]. Peer and spousal support have also been recognized as influential in improving adherence [34]. Telemedicine has emerged as a valuable tool in monitoring and enhancing CPAP compliance. Innovative approaches like remote surveillance and automated communication systems have effectively improved adherence and addressed real-time treatment challenges [34].

### 7.6. Long-Term Impact of ASV Therapy

The effectiveness of adaptive servo-ventilation (ASV) in managing HF patients with SDB has been evaluated through various studies (Table 1), revealing complex outcomes that necessitate a cautious approach in clinical practice. The Treatment of Predominant Central Sleep Apnea by Adaptive Servo Ventilation in Patients with Heart Failure (2015 SERVE-HF) study enrolled 1325 patients who had heart failure with reduced ejection fraction and an AHI of ≥15 or more events per hour, with a predominance of central sleep apnea events [35]. Participants were randomized to receive either guideline-based medical treatment with ASV or guideline-based medical therapy alone. The primary endpoint analyzed was a composite of death from any cause and life-saving cardiovascular or unplanned hospitalization for worsening heart failure [35]. At 12 months, the mean AHI in the ASV group was significantly reduced to 6.6 events per hour [35]. However, the incidence of the primary endpoint did not differ significantly between the ASV group and the control group (54.1% and 50.8%, respectively; hazard ratio 1.13; 95% CI, 0.97 to 1.31; *p* = 0.10) [35]. All-cause mortality and cardiovascular mortality were significantly higher in the ASV group than in the control group, with hazard ratios for death from any cause and cardiovascular death being 1.28 (95% CI, 1.06 to 1.55; *p* = 0.01) and 1.34 (95% CI, 1.09 to 1.65; *p* = 0.006), respectively [35]. These findings were unexpected and suggest that central sleep apnea may have compensatory effects in heart failure that are disrupted by ASV or PAP therapy, which may have adverse effects on cardiac function in a subset of patients.

Further insights come from the 2022 FACE study, a European, multicenter, prospective observational cohort trial that assessed the impact of ASV on morbidity and mortality in HF patients with SDB over a three-month follow-up period. Enrolling 503 diverse participants, predominantly elderly males, the study utilized latent class analysis (LCA) to categorize the individuals into six distinct clusters based on variations in left ventricular ejection fraction (LVEF), SDB types, age, comorbidities and their adherence to ASV therapy [36]. The data from the FACE study indicate that ASV’s effectiveness was not uniformly distributed across all patient profiles. For example, cluster 1, which consisted of patients with severe HF and predominant CSA, exhibited the highest rate of adverse outcomes despite treatment [36]. This suggests that ASV may be less effective or possibly even detrimental in specific HF profiles, particularly those with severe systolic dysfunction and advanced CSA, paralleling concerns raised in previous studies such as the SERVE-HF trial. Among the six clusters identified through LCA, clusters characterized by patients with milder forms of HF, such as those with preserved or mid-range left ventricular ejection fraction (LVEF), and less severe SDB patterns demonstrated the most beneficial responses to ASV, showing trends towards fewer hospitalizations and cardiovascular interventions [36]. This suggests that ASV may be particularly effective in stabilizing patients with a less advanced disease state, enhancing their quality of life and potentially extending survival. Ongoing research and longer-term follow-up within the FACE study will be crucial to validate these initial observations and refine ASV application guidelines. This will provide a more precise roadmap for clinicians to predict which patients will benefit most from ASV therapy.

## 8. Sleep Apnea in Heart Failure: Evaluating Morbidity and Mortality

### 8.1. Mortality Trends in HF Patients with Untreated Moderate to Severe OSA

A prospective observational epidemiologic study conducted at the Heart Failure Clinic of Mount Sinai in Toronto between 1997 and 2004 looked at the mortality rate of HF patients with untreated moderate to severe OSA compared with HF patients with mild to no sleep apnea (M-NSA). The study involved a cohort of 164 HF patients, all of whom underwent polysomnography to determine their classification based on AHI: either as part of the M-NSA group (AHI < 15/h of sleep) or the moderate/severe OSA group (AHI ≥ 15/h of sleep) [37]. Over a mean follow-up period of 2.9 ± 2.2 years, extending up to a maximum of 7.3 years, the study observed a significantly higher mortality rate among the 37 untreated severe/moderate OSA patients compared with the 113 M-NSA patients, with rates of 8.7 vs. 4.2 deaths per 100 patient years, respectively, after adjusting for confounding factors such as left ventricular ejection fraction, NYHA functional class, and age [37]. This significant difference emphasizes the independent risk that untreated moderate/severe OSA poses for increased mortality in HF patients.

### 8.2. Overlap Syndrome

While the general mortality trends associated with untreated moderate to severe OSA among HF patients highlight significant health risks, the situation becomes even more complex when OSA coexists with other chronic respiratory conditions. The study “Mortality in Obstructive Sleep Apnea Syndrome (OSAS) and Overlap Syndrome (OS): The Role of Nocturnal Hypoxemia and CPAP Compliance” highlights the heightened mortality risks in patients with overlap syndrome—defined as the coexistence of OSAS and chronic obstructive pulmonary disease (COPD)—compared with those with only OSAS [38]. This study from 2005 to 2023 revealed a statistically significant increase in mortality rates for OS patients (*p* < 0.001), emphasizing the critical impact of combined respiratory disorders [38]. Key findings indicated that nocturnal hypoxemia and low CPAP adherence are major risk factors, with nocturnal hypoxemia consistently predicting all-cause mortality [38]. Age greater than 65 years and markedly reduced forced expiratory volume in one second (FEV1) also emerged as significant predictors of adverse outcomes in OS patients, with reduced FEV1 correlating with a tenfold increased mortality risk (OR = 10.18, 95% CI 2.32–44.68, *p* = 0.002) [38]. The study emphasized the need for comprehensive management strategies, including optimized CPAP settings and pulmonary rehabilitation, to improve outcomes and quality of life for patients with overlap syndrome.

### 8.3. OSA Symptom Subtypes and Their Impact on Heart Failure Risk

To further understand the diverse impacts of OSA on heart failure, it is essential to explore how different OSA symptom subtypes influence the risk and progression of heart failure. The Sleep Heart Health Study (SHHS) was a multicenter prospective community-based cohort study of 1207 patients with OSA (AHI > 15 events/h) and used an LCA to identify four OSA symptom subtypes: disturbed sleep, minimally symptomatic, moderately sleepy, and excessively sleepy [39]. The study found that the significantly sleepy cohort displayed a threefold increased risk of HF compared with the other subtypes and higher hazard ratios (1.7–2.4) for cardiovascular diseases, coronary heart disease, and HF [39].

### 8.4. Linking OSA Severity to Cardiovascular Outcomes

There is a growing recognition of the interplay between OSA severity and cardiovascular disease (CVD) outcomes. Exploring this relationship requires looking at several physiological and genomic variables. The hypoxic burden, signifying the extent and severity of oxygen deprivation during sleep, has been closely linked with cardiovascular morbidity [40]. Additionally, the total sleep time with SpO_2_ below 90% (TST90) has emerged as a vital mortality predictor, particularly in OSA patients with co-existing cardiac conditions [40]. Numerous research studies have indicated that the metrics of overnight hypoxemia are more effective for predicting mortality than the AHI [41]. A study looking at over 10,000 Canadian patients who were followed for 68 months showed that AHI did not predict mortality, but when nighttime oxygen saturation dropped below 90% for as little as nine minutes, the mortality rate increased by 58% [41]. Another large-scale study of 10,000 individuals who were followed for 5.3 years determined that significant nocturnal hypoxemia doubled the risk of sudden cardiac death, even after adjusting for confounding variables [41]. Nocturnal heart rate variability, indicative of the autonomic system’s response to respiratory events, has become a key marker of cardiac health [40]. This has been supported by a large polysomnography study demonstrating a 17-fold increase in atrial and ventricular arrhythmia following nighttime apnea or hypopnea episodes [31]. The sleep arousal burden, reflecting the frequency and intensity of sleep disturbances, significantly affects long-term cardiovascular health [40]. In addition to these clinical factors, recent genomic studies have identified specific microRNAs altered in OSA, which offer potential as biomarkers for OSA-related CVD [40]. In light of these findings, a nuanced approach that meticulously considers OSA severity, physiological impacts, and genetic markers is crucial for effectively addressing the cardiovascular risks associated with OSA.

## 9. A Comprehensive Approach to Sleep Apnea: Diagnosis and Management

### 9.1. Current Methods of Diagnosing Sleep Apnea

Diagnosing sleep apnea involves various techniques, with polysomnography (PSG) at the forefront due to its comprehensive assessment capabilities. PSG monitors multiple physiological parameters, including brainwave activity, eye movements, muscle activity, heart rate, respiratory effort, airflow, and blood oxygen levels during sleep [42]. This multi-faceted approach makes PSG invaluable for diagnosing various forms of sleep apnea, including the differentiation between OSA and CSA. However, the requirement for an overnight stay in a specialized sleep clinic and the high cost associated with PSG limit its accessibility for many patients [43]. Home-sleep apnea testing (HSAT) provides a more accessible alternative, particularly for diagnosing OSA. HSAT devices in patients’ homes typically measure airflow, respiratory effort, and oxygen saturation [42]. HSAT devices are less intrusive and more cost-effective than PSG but have limitations in accurately diagnosing CSA due to their reduced sensitivity in detecting the absence of respiratory effort characteristic of CSA, often necessitating a follow-up with PSG to confirm a diagnosis, especially in settings where access to specialized sleep clinics is limited.

### 9.2. Advancements in Sleep Apnea Diagnosis through Technology

Additionally, newer diagnostic modalities are emerging to improve the detection and management of sleep apnea. These include wearable devices that monitor physiological parameters such as heart rate variability and oxygen saturation through less invasive means, providing continuous data over multiple nights. This technology could offer a more patient-friendly approach to diagnosing sleep apnea. Furthermore, advancements in software algorithms integrating machine learning (ML) applied to data from HSAT and other wearable devices are improving the ability to differentiate between OSA and CSA. ML models utilizing data from electrocardiograms, pulse oximetry, and sound signals have demonstrated remarkable efficacy in diagnosing sleep apnea, with some models achieving an accuracy rate of 97.1% and a specificity of 100% [43]. 

**Figure 6:**
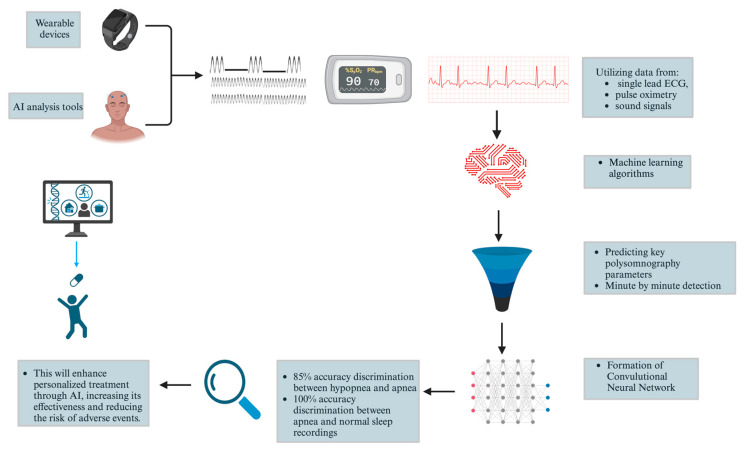
Health monitoring process using wearable devices integrated with AI analysis tools. Data from a single-lead ECG, pulse oximetry, and sound signals are fed into a computer that uses machine learning algorithms to predict key polysomnography parameters and perform minute-by-minute detection. The convolutional neural network formed can achieve an 85% accuracy rate in distinguishing between hypopnea and apnea and a 100% accuracy rate in differentiating between apnea and normal sleep recordings, enabling early detection and intervention.

Furthermore, these models have shown proficiency in predicting key polysomnographic parameters [43]. Notably, a convolutional neural network (CNN) using single-lead ECG data yielded a per-recording classification accuracy of 97.1%, specificity of 100%, and sensitivity of 95.7%, far surpassing several traditional diagnostic approaches [43]. In another innovative approach, an automated algorithm for sleep apnea detection based on single-lead ECG data demonstrated accuracies of 85% in minute-by-minute detection of both hypopnea and apnea. It could discriminate between apnea and normal recordings with 100% accuracy [43] (Figure 6).

### 9.3. Emerging Non-CPAP Therapies for OSA Management

**Figure 7:**
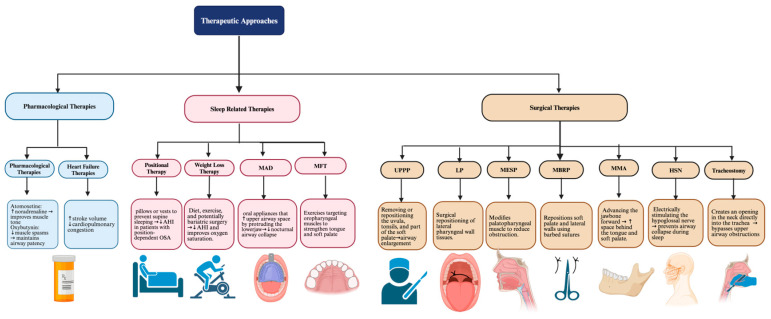
Summary of the major therapies for sleep-disordered breathing, spotlighting the roles of pharmacological treatments, sleep-related therapies, and surgical therapies. The figure outlines the benefits of atomoxetine and oxybutynin for patients who find CPAP machines challenging, examines how heart failure medications reduce central sleep apnea symptoms, and emphasizes the value of positional therapy and weight loss in managing obstructive sleep apnea non-invasively. It also highlights mandibular advancement devices (MADs) as a preferred option for patients with mild to moderate OSA and reviews some of the latest surgical techniques.

Exploring novel therapeutic avenues for OSA has revealed significant potential in various non-CPAP interventions (Figure 7). Positional therapy and weight loss programs have emerged as effective strategies, particularly in patients whose OSA severity is influenced by body position or obesity [44]. Pharmacological interventions like atomoxetine and oxybutynin target the pharyngeal dilator muscles to maintain airway patency during sleep. A meta-analysis of randomized controlled trials demonstrated that this pharmacotherapy reduced AHI and improved oxygen saturation levels but only modestly [45]. In addition, the side effects of these pharmacological interventions, such as dry mouth, fatigue, and gastrointestinal discomfort, pose challenges to patient compliance and long-term usability.

Myofunctional therapy (MFT) is also emerging as a noninvasive and effective treatment for obstructive sleep apnea (OSA). It targets the oropharyngeal muscles through specific exercises that strengthen the tongue and soft palate [46]. This therapy enhances muscle tone and stability, reducing airway collapse during sleep and decreasing AHI and snoring frequency [46]. It is particularly beneficial for patients who are non-compliant with CPAP therapy; MFT improves sleep quality and reduces daytime sleepiness without significant side effects.

Mandibular advancement devices (MADs), traditionally second-line therapy, are now finding increased utility as a primary approach for mild to moderate symptomatic OSA. Their effectiveness in reducing AHI is less than that of CPAP. Still, their utility is bolstered by greater patient adherence and a mean disease alleviation rate comparable to that of CPAP [44]. This balance of efficacy and patient preference makes them a viable alternative to CPAP therapy despite practical limitations like dental contraindications and customization challenges [44].

Surgical interventions for OSA play a role by targeting anatomical obstructions in the airway. Among these, uvulopalatopharyngoplasty (UPPP) is designed to enlarge the airway by removing or repositioning tissues such as the uvula, tonsils, and part of the soft palate [47]. Although traditionally popular, uvulopalatopharyngoplasty (UPPP) has seen a decline in usage due to its variable success rates and post-operative complications, such as velopharyngeal insufficiency and foreign body sensation [48]. Instead, lateral pharyngoplasty (LP) has become a more practical alternative, particularly for patients with lateral pharyngeal wall collapse [48]. Studies have shown that while UPPP and LP significantly improve the AHI and oxygen saturation, LP tends to have better outcomes and fewer complications, making it a preferable option in many cases.

Additionally, modified expansion sphincter pharyngoplasty (MESP) and modified barbed reposition pharyngoplasty (MBRP) have been developed as effective surgical techniques for addressing retropalatal collapse in OSA patients [49]. Drug-induced sleep endoscopy (DISE) is often utilized to precisely identify the sites of airway collapse, guiding the selection of the most appropriate surgical intervention [49]. MESP involves modifying the palatopharyngeal muscle to reduce oropharyngeal obstruction and improve airflow [49]. MBRP utilizes a barbed suture technique to reposition and stabilize the soft palate and lateral pharyngeal walls, thus enlarging the airway with minimal tissue removal and reduced postoperative discomfort [49]. In a comparative study, both MESP and MBRP showed significant reductions in AHI and oropharyngeal obstruction and had high success rates—90% for MESP and 80% for MBRP, according to Sher’s criteria (surgical success for treating obstructive sleep apnea as a postoperative reduction of the apnea–hypopnea index (AHI) by more than 50% and a postoperative AHI of less than 20 events per hour.) [50]. Notably, MBRP was associated with less postoperative pain and faster recovery, attributed to its minimally invasive nature and the absence of mucosal damage [49].

Maxillomandibular advancement (MMA) surgery works by surgically advancing the jawbone forward, significantly enlarging the airway space behind the tongue and soft palate. This method effectively reduces OSA severity and offers considerable improvements in nocturnal function [47]. Hypoglossal nerve stimulation (HNS) represents a contemporary, less invasive technique that maintains airway patency by electrically stimulating the hypoglossal nerve, which prevents airway collapse during sleep [47]. Tracheostomy, considered a last-resort option due to its invasiveness and life-altering implications, effectively bypasses upper airway obstructions, ensuring unimpeded ventilation during sleep [47]. Each surgical strategy, from the intricate tissue reconfiguration in UPPP and the structural airway expansion in MMA to the targeted neural activation in HNS and the definitive airway clearance in tracheostomy, presents unique advantages and disadvantages, necessitating careful consideration for integration into the holistic care plan for HF patients with OSA.

### 9.4. Management of CSA in HF

Treatment options for CSA are diverse, with each approach backed by varying degrees of evidence and associated with specific advantages and disadvantages. PAP therapies, such as CPAP and ASV, are foundational in managing CSA. CPAP is particularly beneficial, improving ejection fraction and transplant-free survival in patients with concomitant HF and CSA [6]. Despite its effectiveness in stabilizing the upper airway, improving sleep quality, and enhancing daytime alertness, some patients find CPAP cumbersome and uncomfortable, leading to poor adherence. ASV, which variably adjusts pressure support to normalize breathing patterns, is superior in specific patient subsets, especially those with heart failure. However, its use is contraindicated in patients with symptomatic heart failure and reduced ejection fraction, due to increased mortality risk, as evidenced by the SERVE-HF trial [50]. Supplemental nasal oxygen is recognized as a helpful intervention in managing CSA, especially when it is associated with conditions such as heart failure. The therapeutic benefit of nasal oxygen in CSA is primarily due to its capacity to stabilize the patient’s breathing pattern by mitigating hypoxemia and reducing the magnitude of post-apneic ventilatory overshoot [50]. Physiologically, administering supplemental oxygen can lessen the severity of nocturnal hypoxemia and thus dampen the ventilatory drive fluctuations that typically provoke CSA episodes. The American Academy of Sleep Medicine recommends using nocturnal oxygen as a standard treatment for CSA related to heart failure, emphasizing its role in managing this complex condition [50]. Despite the positive effects, the long-term impact and optimal administration strategies of supplemental oxygen in CSA patients remain areas of active research and clinical evaluation.

Since CSA is intricately linked with systolic HF, the management strategy also involves pharmacological therapy with diuretics, angiotensin-converting enzyme inhibitors, β-blockers, and cardiac pacing when indicated [51]. Alternatives such as acetazolamide, theophylline, and nocturnal nasal oxygen can be considered when conventional treatments do not work [51]. Acetazolamide, a carbonic anhydrase inhibitor, works by inducing mild metabolic acidosis, which stimulates the chemoreceptors in the body to enhance respiratory drive [50]. Despite its effectiveness in some cases of CSA, particularly those associated with altitude sickness or conditions that disrupt normal respiratory control, the use of acetazolamide is limited due to side effects such as numbness, tingling, and potential metabolic disturbances, including alterations in electrolyte balance and acid-base status [50]. These side effects often relegate acetazolamide to a second-line option. Theophylline, a phosphodiesterase inhibitor with a long history of use in respiratory diseases, particularly asthma and chronic obstructive pulmonary disease, has a stimulatory effect on the respiratory centers in the brain. However, its utility in treating CSA is constrained by its narrow therapeutic window and a range of adverse effects, such as palpitations, tremors, and insomnia, necessitating careful monitoring of blood levels to avoid toxicity [50].

Emerging therapies such as phrenic nerve stimulation, which involves the surgical implantation of a device that rhythmically stimulates the phrenic nerve to activate the diaphragm, have shown promise [50]. Early results indicate significant improvements in AHI and patient quality of life, although surgical risks and the need for procedural expertise limit its widespread adoption.

Overall, the choice of therapy for CSA should be guided by individual patient characteristics, including the presence of comorbid conditions, particularly heart failure, patient tolerance, and preferences, thereby necessitating a personalized approach to treatment.

## 10. Conclusions

In this comprehensive review, the authors have sought to elucidate the intricate interplay between SDB and HF, providing a thorough exploration of the pathophysiological mechanisms and therapeutic implications. This review effectively summarizes the current body of knowledge and underscores the urgent need for enhanced diagnostic tools and treatment modalities, including paying particular attention to the efficacy of therapies such as CPAP and ASV.

While this review is comprehensive and informative, its scope is primarily focused on existing literature and secondary data analyses, which naturally constrains its breadth to the studies selected for inclusion. While effective in providing detailed insights, this selective focus may only capture part of the spectrum of emerging research in this rapidly evolving field. Moreover, the discussion on the practical challenges of patient compliance and the real-world application of treatment strategies is limited. Addressing these aspects could enhance the practical utility of the review for clinical practitioners.

## 11. Future Research Directions in SDB and Heart Failure

SDB such as OSA and CSA adversely affects HF through an increased cardiac load, structural heart changes, and intermittent hypoxia. Recognizing OSA and its diverse phenotypes requires a personalized approach to treatment. Clinically, OSA presents variably, ranging from asymptomatic cases to those with severe sleep disturbances and excessive daytime sleepiness [52]. Anatomically, it is characterized by a spectrum of structural variations, including craniofacial and upper airway anatomical differences that significantly impact its manifestation and treatment response [52]. Polysomnographic phenotyping further reveals distinct patterns in sleep disruptions, from variations in AHI to differences in sleep architecture [51]. The burgeoning field of transcriptomic profiling further bolsters the role of microRNAs (miRNAs) as novel biomarkers in OSA [52]. By modulating gene expression, these regulatory molecules hold promise in elucidating the distinct phenotypic presentations of OSA, particularly those linked with cardiovascular comorbidities [53]. Emerging evidence suggests that specific miRNA profiles may predict responses to conventional treatments like CPAP in OSA patients, paving the way for more effective, individualized therapeutic strategies [52]. These innovations represent a holistic approach to sleep-disordered breathing, integrating cutting-edge research and clinical insights to optimize care for HF patients with OSA and CSA.

Future research could further delineate the role of miRNAs in OSA, exploring how these biomarkers can be used to predict treatment outcomes and develop new therapeutic targets. Additionally, studies could investigate the genetic underpinnings of OSA and CSA, identifying genetic markers contributing to the variability in clinical presentations and treatment responses. Advanced imaging techniques could be employed better to understand the anatomical differences in patients with OSA, leading to more tailored interventions. Another promising area is the development of wearable technologies for continuous monitoring of sleep and respiratory patterns, offering a non-invasive solution to manage and predict exacerbations in real time. Collaborative research across disciplines could also explore the intersection of behavioral, psychological, and physiological factors in the management of OSA and CSA, aiming to develop comprehensive care models that address the multifaceted nature of these disorders.

## Figures and Tables

**Table 1:** Summary of studies exploring the impact of sleep-disordered breathing therapies on heart failure (HF) patients, emphasizing the role of continuous positive airway pressure (CPAP) therapy. The table documents significant improvements in cardiac structure, function, and patient symptoms with CPAP. It also discusses the variable effectiveness of CPAP in different patient populations, noting particularly its limited impact on reducing cardiovascular events and mixed effects among obese patients. Additionally, it discusses the Canadian Continuous Positive Airway Pressure for Central Sleep Apnea and Heart Failure (CANPAP) trial outcomes, indicating the benefits of CPAP yet its failure to alter heart transplant-free survival rates significantly. It also reviews innovative therapies like adaptive servo-ventilation (ASV), highlighting their potential benefits but addressing concerns over increased mortality rates in certain groups. The summary emphasizes the complexity of treating sleep-disordered breathing in HF patients and the necessity for personalized treatment approaches.

Reference/Study	Patient Group	Key Findings
Hall et al. [25]	OSA and HF	CPAP treatment significantly improved myocardial sympathetic nerve function, as evidenced by increased hydroxyephedrine retention. It also led to improvements in work metabolic index and systolic blood pressure in patients with apnea–hypopnea index > 20 events per hour.
Shivalkar et al. [26]	Severe OSA without cardiac or pulmonary disease	CPAP treatment for six months led to significant improvements in symptoms, hemodynamics, and both left and right ventricular morphology and function.
Xu et al. [27]	OSA stratified by obesity	Significant differences in cardiac structure and function between non-obese and obese OSA patients. CPAP therapy reduced NT-proBNP levels in non-obese OSA patients, indicating improved cardiac function, but had mixed effects in obese patients.
Mehra et al. [28]	Moderate-to-severe OSA and cardiovascular disease.	The SAVE trial showed that CPAP was not associated with a statistically significant reduction in cardiovascular events.
Holt et al. [29]	Newly diagnosed sleep apnea in Denmark from 2000 to 2012	Of the 40,485 patients in Denmark who were diagnosed with sleep apnea from 2000 to 2012, it was found that only 45.2% of these patients were adherent to CPAP therapy. Furthermore, there was no notable variance in adherence to heart failure medication between those who adhered to CPAP therapy and those who did not.
McEvoy et al. [30]	Moderate-to-severe OSA and existing cardiovascular disease	CPAP therapy showed notable benefits in reducing symptoms of OSA, such as snoring and daytime sleepiness, and in enhancing quality of life and mood among participants. However, CPAP therapy did not prevent cardiovascular events in this patient population.
Sommerfield et al. [31]	OSA and HF	Patients with concomitant heart failure and OSA were at a significantly elevated risk of hospital readmission within 30 and 90 days following discharge compared with their counterparts without OSA.
Malhotra et al. [32]	Adherent OSA, intermediate adherent OSA, and nonadherent OSA	Adherent patients encountered significantly fewer composite hospital visits and lower composite visit costs compared with their matched nonadherent and intermediate adherent counterparts.
Lyons et al. [33]	CSR-CSA and HF	The Canadian Continuous Positive Airway Pressure for CSA and HF (CANPAP) trial observed that treating CSA with CPAP for two years improved nocturnal oxygenation, LVEF, and exercise capacity. However, CPAP failed to suppress the AHI below the trial entry threshold in 43% of patients and did not affect heart transplant-free survival.
Donovan et al. [34]	Severe OSA	The study explored enhancements in CPAP therapy using BiPAP and EPR technologies aimed at improving patient comfort and adherence. BiPAP devices were noted for effectively reducing mean airway pressure and facilitating easier breathing for patients with severe OSA. EPR technology was highlighted for its role in improving patient comfort and potentially increased adherence rates.
Cowie et al. [35]	CSR-CSA and HFrEF	The SERVE-HF trial showed that ASV did not significantly improve the primary composite endpoint of all-cause mortality, life-saving cardiovascular intervention, or unplanned hospitalization for worsening chronic HF. More concerning was the observation of increased all-cause and cardiovascular mortality in the ASV group compared to the control group.
Tamisier et al. [36]	Patients with HF categorized into different subgroups based on clinical characteristics, predominant CSA ± OSA, and varying degrees of LVEF (HFrEF, HFmrEF, HFpEF) indicated for ASV therapy	The FACE study 3-month follow-up showed the primary outcome events were significantly higher in cluster 1 (men, low LVEF, severe HF, CSA; 13.9% vs. 1.5–5% in other clusters, *p* < 0.01). This indicates that homogeneous patient clusters represent clinically relevant subgroups for SDB management in HF, potentially leading to personalized therapy approaches.
